# A systemic review of taxanes and their side effects in metastatic breast cancer

**DOI:** 10.3389/fonc.2022.940239

**Published:** 2022-10-11

**Authors:** Jiun-I. Lai, Ta-Chung Chao, Chun-Yu Liu, Chi-Cheng Huang, Ling-Ming Tseng

**Affiliations:** ^1^ Division of Medical Oncology, Department of Oncology, Taipei Veterans General Hospital, Taipei, Taiwan; ^2^ Center of Immuno-Oncology, Department of Oncology, Taipei Veterans General Hospital, Taipei, Taiwan; ^3^ Institute of Clinical Medicine, School of Medicine, National Yang Ming Chiao Tung University, Taipei, Taiwan; ^4^ Comprehensive Breast Health Center, Department of Surgery, Taipei Veterans General Hospital, Taipei, Taiwan; ^5^ Faculty of Medicine, School of Medicine, National Yang Ming Chiao Tung University, Taipei, Taiwan; ^6^ Division of Transfusion Medicine, Department of Medicine, Taipei Veterans General Hospital, Taipei, Taiwan; ^7^ Division of Experimental Surgery, Department of Surgery, Taipei Veterans General Hospital, Taipei, Taiwan; ^8^ School of Public Health, College of Public Health, National Taiwan University, Taipei, Taiwan; ^9^ Division of General Surgery, Department of Surgery, Taipei Veterans General Hospital, Taipei, Taiwan

**Keywords:** docetaxel, paclitaxel, breast cancer, adverse event, systemic review

## Abstract

Taxanes-containing chemotherapy constitutes an essential backbone for both early and metastatic breast cancer (mBC). However, the two major taxane drugs—paclitaxel and docetaxel—have distinct safety profiles. In this review, we summarize the safety outcome and management following treatment with both taxanes from selected clinical trials. We utilized PubMed to perform literature search before April 2021. Five phase III randomized controlled trials with reports of individual taxane adverse events (AEs) were included in this review. Grade 3/4 AEs were summarized and discussed extensively. The rates of grade 3/4 neutropenia were higher with docetaxel than with paclitaxel. For non-hematologic grade 3/4 AEs, peripheral neuropathy was more frequent with paclitaxel while fluid retention was more frequent with docetaxel. Compared to paclitaxel, docetaxel had a higher rate of grade 3/4 gastrointestinal AEs. Grade 3/4 myalgia were generally comparable between the two taxanes. Except for neutropenia, the incidence rate of grade 3/4 AEs of taxanes was generally manageable. Peripheral neuropathy was more common with paclitaxel while grade 3/4 neutropenia was more common with docetaxel.

## 1 Introduction

Breast cancer ranks among the top two most commonly diagnosed cancer worldwide. Despite a significant improvement in breast cancer management and availability of novel drugs in the past two decades, the mortality rate of breast cancer patients remains high (1, 2). An estimated 1,671,149 people were diagnosed with breast cancer, while 521,907 people die of breast cancer every year worldwide (3).

Taxanes, especially paclitaxel and docetaxelare chemotherapeutic agents commonly used to treat breast cancer. Paclitaxel was discovered in the extracts of the bark tissue of Pacific yew (*Taxus brevifolia*) in an NCI program in 1963, while docetaxel was extracted from the European yew (*Taxus baccata*) in a search for taxane drugs to improve survival (4, 5). Taxanes are approved by FDA and EMA for different cancers, including breast cancer, lung cancer, gastric cancer, and ovarian cancer (6).

Taxanes constitute one of the major chemotherapy backbones for breast cancer. In early breast cancer, the addition of taxanes to anthracyclines demonstrates a significant reduction in recurrence risk and risk of death in the CALGB 9344 and NSABP B30 trials (7, 8). In the metastatic setting, both paclitaxel and docetaxel have demonstrated significant activity in HER2 mBC (9, 10) and are both recommended as standard regimens by NCCN guidelines (ref to be provided). In HER2 mBC, the preferred 1^st^ line treatment is dual HER2 blockade by trastuzumab plus pertuzumab, combined with docetaxel as a standard chemotherapy partner (11), although paclitaxel and nab-paclitaxel can be substituted (12). Animal studies showed that taxanes have synergistic therapeutic effect when used in combination with trastuzumab, with docetaxel showing direct NK Group 2 member D (NKG2D) receptors upregulation, which increases the antibody-dependent cellular cytotoxicity (ADCC) effects delivered *via* trastuzumab (13, 14). Current guidelines establish taxanes as an indispensable therapeutic option that is active and well-tolerated in both early and metastatic BC, highlighting the importance for medical and surgical oncologists to be familiarized with the efficacy and safety profiles of this group of chemotherapeutics.

The administration of taxanes can be a double-edged sword that requires additional considerations. In the past twenty years, many studies have focused on the discussion of taxane-related side effects. Given that more than half of the patients have their first breast cancer diagnosis over the age of 65, adequate management of symptoms associated with these agents is needed to improve quality of life (QoL), especially for patients with advanced disease (12, 15–17), who often may require dose reductions (4, 18).

Paclitaxel and docetaxel have a distinct safety profile, and require different premedications before use (19, 20). Hence, in this study, we aimed to comprehensively compare grade 3/4 AEs associated with paclitaxel and docetaxel tin published randomized controlled trials (RCTs), with a special focus on the management of adverse events. Our study aimed to systemically analyze the clinical applicability and ease of use of the two major taxanes, paclitaxel and docetaxel, in order to improve insight and knowledge of oncologists prescribing these regimens.

## 2 Methods

We adhered to Preferred Reporting Items for Systematic reviews and Meta-Analyses (PRISMA) guidelines for conducting and reporting this systematic review. We aimed to compare the safety profile between the paclitaxel and the docetaxel.

### 2.1 Search strategy

To conduct this review, two authors (J.I.L, T.C.C) independently screened the articles in PubMed to retrieve randomized clinical trials published in PubMed before April 2021. Patients of interest were defined as patients with a diagnosed breast cancer. Keywords used to search appropriate studies included ((cancer[Title]) OR (malig*[Title]) OR (neopla*[Title]) OR (carcin*[Title])) AND ((docetaxel[Title/Abstract]) AND (paclitaxel[Title/Abstract])) AND ((trial[Title]) OR (study[Title])) AND (breast).

To be eligible, studies retrieved from PubMed, had to report grade 3/4 AEs of individual taxanes, either in the text or in supplementary appendix. To minimize cross-trial comparison conclusions as a potential source of bias, we only included phase 3 randomized trials that had both paclitaxel and docetaxel in the same trial (in different cohorts) in order to allow a meaningful comparison of AEs. Full-text articles were thoroughly examined by two independent authors to identify qualified articles and further discussed to resolve the discrepancies in the study selection. Grade 3/4 AEs from the included studies were extracted and summarized to compare the safety profile of paclitaxel and docetaxel.

## 3 Result

### 3.1 Study selection

**Figure 1:**
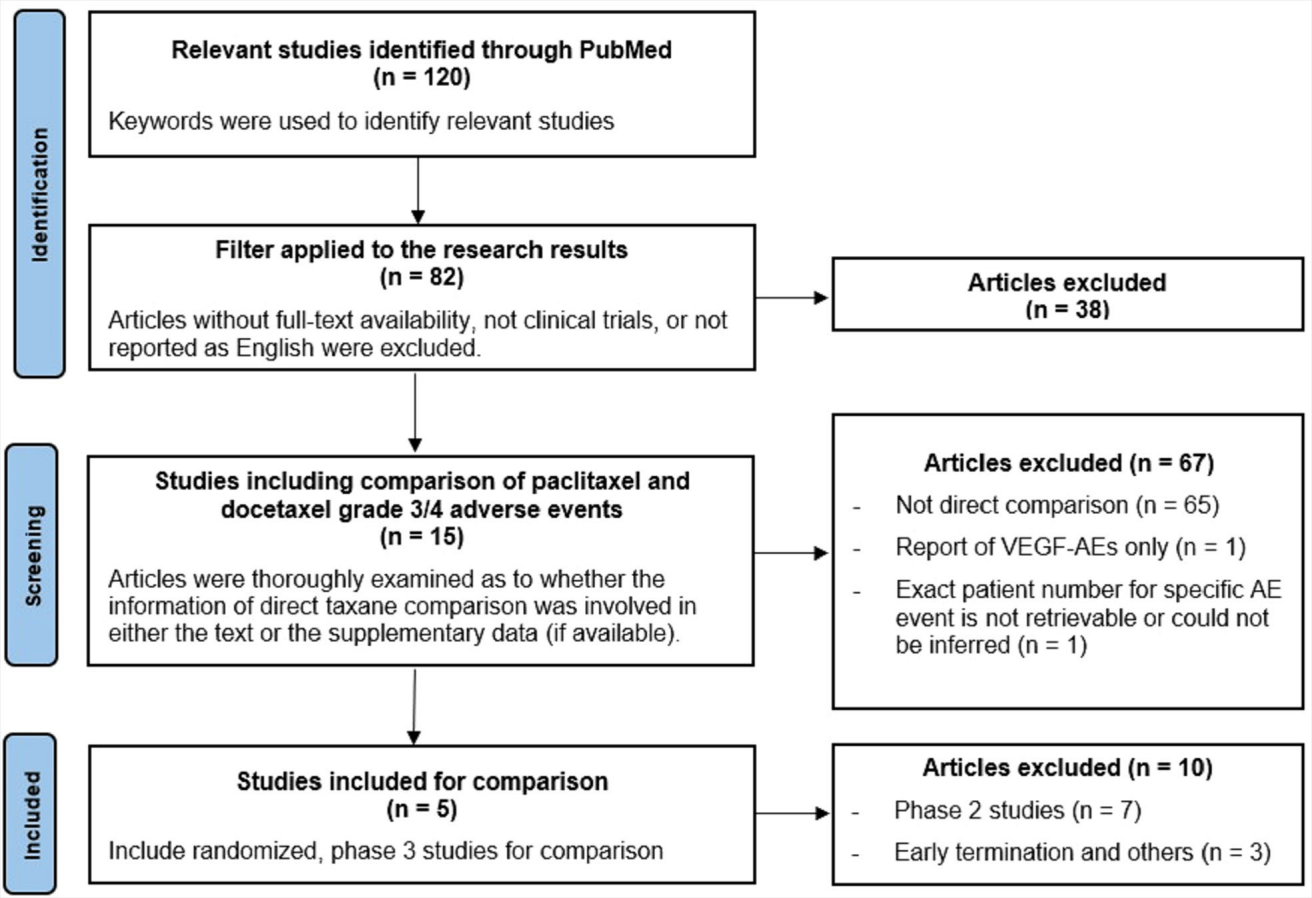
Study searching diagram based on the PRISMA guideline.

**Figure 1** shows the flow diagram of study selection. Our criteria for selecting clinical trials were mainly based on evaluability of reported adverse effects when paclitaxel or docetaxel was contained in the treatment regimen. A total of 120 studies were identified. Articles without full-text availability, reports not related to clinical trials, or not reported in English were excluded. The full-text of each article was thoroughly examined to determine if grade 3/4 AEs of taxanes were reported either in the text or in the supplementary appendix (if available). Overall 15 articles were selected, and five of them were randomized phase 3 studies satisfying inclusion in our study.

### 3.2 Study characteristics

**Table 1** presents the five studies included in our analysis comparing the grade 3/4 AEs of paclitaxel and docetaxel. Swain et al. (22) had an enrollment of the most patients (4,894 patients), followed by 1060 by Watanabe et al. (23) and 601 patients by Kelly et al. (21). Jones et al. (5) recruited 449 patients, while Cassier et al. (17) enrolled 210 patients with quality of life as primary endpoint.

**Table 1:** Characteristics of the five eligible trials.

Study					Regimen of taxanes						Combined Agent	Premedication	GCSF prophylaxis
Author, year	Phase	Trial Design	PatientPopulation	Arms	dose [mg/m^2^], frequency			Cycle		Patient NumberCohort (Safety)			
								Planned	Median				
Jones et al., 2005 (5)	3	Randomized Open-label Multi-center	Locally advanced, Metastatic	2	D	100	Q3W	Until PD	6	225 (222)	Nil	Dexamethasone	Primary: Not specified Secondary: Yes
					P	175	Q3W		4	224 (222)		Dexamethasone + Diphenhydramine + Ranitidine/Cimetidine	
Cassier et al., 2008 (17)	3	Randomized Open-label Multi-center	Metastatic	2	D	75	Q3W	8	8	107 (72)	Doxorubicin (last four cycles)	Not specified	Primary: (at baseline) D: 9.3%; P 10.7%
					P	175	Q3W	8	8	103 (77)			
Kelly et al., 2012 (21)	3	Randomized Open-label Single-center	Adjuvant	2	D	75	Q3W	4	NA	300 (292)	Capecitabine	Not specified	Not specified
					P	80	QW	12		301 (297)	Nil		
Swain et al., 2013 (22)	3	Randomized Multi-center	Adjuvant	3	D	75	Q3W	6	NA	1630 (1607)	doxorubicin + cyclophosphamide	Not specified	Primary prophylaxis for all patients
					P	175	Q2W	4		1634 (1623)			
					P	175	Q2W	4		1630 (1612)			
Watanabe et al., 2017 (23)	3	Randomized Open-label Multi-center	Adjuvant	4	D	75	Q3W	4	NA	265 (263)	Doxorubicin + Cyclophosphamide	Not specified	Not specified
						75	Q3W	8		265 (261)	Nil		
					P	175	Q3W	4		263 (262)	Doxorubicin + Cyclophosphamide		
						175	Q3W	8		267 (263)	Nil		

D, docetaxel; P, paclitaxel.

For Jones et al., there was no combined chemotherapy. For Cassier et al., both groups combined taxane with doxorubicin in the last four cycles. For Kelly et al., the docetaxel group was combined with capecitabine. For Swain et al., all the three groups were combined with doxorubicin and cyclophosphamide. For Watanabe et al., Two of the groups took doxorubicin and cyclophosphamide.

In the docetaxel group, 90% of the patients received preplanned number of treatment cycles (Q3W for 4 cycles); In the paclitaxel group, 86% received preplanned number of treatment cycles (QW for 12 cycles).

NA, not available.

Two dosing schedules, weekly (QW) and tri-weekly (Q3W), were commonly used. The dose of tri-weekly paclitaxel was mainly 175 mg/m^2^. The dose of tri-weekly docetaxel was usually 75 mg/m^2^, except in the Jones’ trial (5), which used a dose of 100 mg/m^2^. Kelly et al. (21) compared tri-weekly docetaxel 75 mg/m^2^ to weekly paclitaxel 80 mg/m^2^. In Swain’s trial (22), docetaxel 75 mg/m^2^ was given every three weeks, while paclitaxel was given at 175 mg/m^2^ every three weeks.

Jones et al. used taxanes in recurrent metastatic breast cancer patients who had been previously treated with an anthracycline-based regimen. Swain et al. reported three cohorts of patients treated with taxanes in combination with doxorubicin and cyclophosphamide. Cassier et al. (17) reported patients receive taxanes (docetaxel or paclitaxel) in combination with doxorubicin for 4 cycles, followed by 4 cycles with the same taxane (either docetaxel or paclitaxel) in monotherapy for another 4 cycles. Kelly et al. (21) combined docetaxel with capecitabine. Watanabe et al. (23) reported four treatment groups: AC (doxorubicin/cyclophosphamide) containing-regimens (AC plus docetaxel or AC plus paclitaxel) versus the AC free-regimens (docetaxel or paclitaxel alone).

In Jones et al. (5), patients received premedication with dexamethasone before docetaxel and diphenhydramine and ranitidine or cimetidine before paclitaxel. For the prevention of neutropenia, Jones et al. used G-CSF as secondary prophylaxis (5), while Cassier et al. reported that around 10% of patients received prophylactic G-CSF (17).

### 3.3 Grade 3/4 adverse events

**Table 2** and **Table 3** summarize the grade 3/4 AEs in the five studies.

**Table 2:** Grade 3 and 4 hematologic adverse events summarized from the included five trials.

Grade 3/4 adverse events	Study	Docetaxel		Paclitaxel	
		Incidence (%)	Regimen	Incidence (%)	Regimen
Neutropenia	Jones et al.	93.3	100 mg/m^2^ Q3W	54.5	175 mg/m^2^ Q3W
	Cassier et al.	27.8	75 mg/m^2^ Q3W	7.8	175 mg/m^2^ Q3W
	Kelly et al.	15.4	75 mg/m^2^ Q3W	0.6	80 mg/m^2^ QW
	Watanabe et al.	6.9	75 mg/m^2^ Q3W	1.9	175 mg/m^2^ Q3W
Leukopenia	Cassier et al.	12.5	75 mg/m^2^ Q3W	3.9	175 mg/m^2^ Q3W
	Watanabe et al.	3.1	75 mg/m^2^ Q3W	0.4	175 mg/m^2^ Q3W
Febrile Neutropenia	Jones et al.	14.9	100 mg/m^2^ Q3W	1.8	175 mg/m^2^ Q3W
	Cassier et al.	2.8	75 mg/m^2^ Q3W	1.3	175 mg/m^2^ Q3W
	Kelly et al.	4.4	75 mg/m^2^ Q3W	0.0	80 mg/m^2^ QW
	Watanabe et al.	8.1	75 mg/m^2^ Q3W	0.4	175 mg/m^2^ Q3W
	Swain et al.	9.0	75 mg/m^2^ Q3W	3.1	175 mg/m^2^ Q2W
Anemia	Cassier et al.	0.0	75 mg/m^2^ Q3W	1.3	175 mg/m^2^ Q3W
	Swain et al.	0.2	75 mg/m^2^ Q3W	1.6	175 mg/m^2^ Q2W
Thrombocytopenia	Jones et al.	4.6	100 mg/m^2^ Q3W	2.8	175 mg/m^2^ Q3W

Jones et al. did not report incidence of 3% or lesser in either treatment group.

Data of adverse events from Cassier et al. were from Cycle 5-8 in the study, when the patients were combined with doxorubicin.

**Table 3:** Grade 3 and 4 non-hematologic adverse events summarized from the included five trials.

Grade 3/4 adverse events	Study	Docetaxel		Paclitaxel	
		Incidence (%)	Regimen	Incidence (%)	Regimen
Peripheral Edema	Jones et al.	6.8	100 mg/m^2^ Q3W	0.5	175 mg/m^2^ Q3W
	Cassier et al.	2.8	75 mg/m^2^ Q3W	0.0	175 mg/m^2^ Q3W
	Kelly et al.	0.0	75 mg/m^2^ Q3W	0.3	80 mg/m^2^ QW
	Watanabe et al.	12.6	75 mg/m^2^ Q3W	0.0	175 mg/m^2^ Q3W
Peripheral Neuropathy	Cassier et al.	0.0	75 mg/m^2^ Q3W	7.8	175 mg/m^2^ Q3W
	Kelly et al.	4.8	75 mg/m^2^ Q3W	2.4	80 mg/m^2^ QW
Neurosensory	Jones et al.	7.2	100 mg/m^2^ Q3W	4.1	175 mg/m^2^ Q3W
	Swain et al.	1.0	75 mg/m^2^ Q3W	6.7	175 mg/m^2^ Q2W
	Watanabe et al.	3.8	75 mg/m^2^ Q3W	5.7	175 mg/m^2^ Q3W
Neuromotor	Jones et al.	5.0	100 mg/m^2^ Q3W	2.3	175 mg/m^2^ Q3W
	Watanabe et al.	1.3	75 mg/m^2^ Q3W	0.4	175 mg/m^2^ Q3W
Nausea	Jones et al.	5.4	100 mg/m^2^ Q3W	2.7	175 mg/m^2^ Q3W
	Kelly et al.	4.1	75 mg/m^2^ Q3W	1.7	80 mg/m^2^ QW
	Swain et al.	3.9	75 mg/m^2^ Q3W	3.2	175 mg/m^2^ Q2W
	Watanabe et al.	1.2	75 mg/m^2^ Q3W	0.4	175 mg/m^2^ Q3W
Vomiting	Jones et al.	3.2	100 mg/m^2^ Q3W	0.0	175 mg/m^2^ Q3W
	Kelly et al.	1.7	75 mg/m^2^ Q3W	0.0	80 mg/m^2^ QW
	Swain et al.	2.8	75 mg/m^2^ Q3W	2.9	175 mg/m^2^ Q2W
	Watanabe et al.	0.8	75 mg/m^2^ Q3W	0.0	175 mg/m^2^ Q3W
Diarrhea	Jones et al.	5.4	100 mg/m^2^ Q3W	0.5	175 mg/m^2^ Q3W
	Kelly et al.	5.8	75 mg/m^2^ Q3W	4.0	80 mg/m^2^ QW
	Swain et al.	6.8	75 mg/m^2^ Q3W	2.2	175 mg/m^2^ Q2W
	Watanabe et al.	1.9	75 mg/m^2^ Q3W	0.4	175 mg/m^2^ Q3W
Constipation	Kelly et al.	2.0	75 mg/m^2^ Q3W	0.7	80 mg/m^2^ QW
	Watanabe et al.	0.4	75 mg/m^2^ Q3W	0.4	175 mg/m^2^ Q3W
Skin Disorders	Jones et al.	4.5	100 mg/m^2^ Q3W	0.0	175 mg/m^2^ Q3W
	Kelly et al.	0.3	75 mg/m^2^ Q3W	1.0	80 mg/m^2^ QW
Mucositis (including stomatitis)	Jones et al.	10.8	100 mg/m^2^ Q3W	0.0	175 mg/m^2^ Q3W
	Cassier et al.	0.0	75 mg/m^2^ Q3W	1.3	175 mg/m^2^ Q3W
	Swain et al.	0.9	75 mg/m^2^ Q3W	0.8	175 mg/m^2^ Q2W
	Kelly et al.	1.7	75 mg/m^2^ Q3W	0.0	80 mg/m^2^ QW
Elevated AST or ALT	Watanabe et al.	0.4	75 mg/m^2^ Q3W	3.1	175 mg/m^2^ Q3W
Elevated Bilirubin	Watanabe et al.	0.0	75 mg/m^2^ Q3W	0.4	175 mg/m^2^ Q3W
Fatigue	Kelly et al.	22.5	75 mg/m^2^ Q3W	8.4	80 mg/m^2^ QW
	Swain et al.	9.2	75 mg/m^2^ Q3W	9.1	175 mg/m^2^ Q2W
	Watanabe et al.	1.9	75 mg/m^2^ Q3W	1.9	175 mg/m^2^ Q3W
Asthenia	Jones et al.	20.7	100 mg/m^2^ Q3W	5.0	175 mg/m^2^ Q3W
	Cassier et al.	5.6	75 mg/m^2^ Q3W	6.5	175 mg/m^2^ Q3W
Arthralgia	Kelly et al.	1.0	75 mg/m^2^ Q3W	0.0	80 mg/m^2^ QW
	Swain et al.	4.1	75 mg/m^2^ Q3W	11.8	175 mg/m^2^ Q2W
	Watanabe et al.	1.6	75 mg/m^2^ Q3W	7.5	175 mg/m^2^ Q3W
Myalgia	Jones et al.	2.7	100 mg/m^2^ Q3W	3.2	175 mg/m^2^ Q3W
	Kelly et al.	10.9	75 mg/m^2^ Q3W	6.4	80 mg/m^2^ QW
	Watanabe et al.	0.8	75 mg/m^2^ Q3W	5.3	175 mg/m^2^ Q3W

Jones et al. did not report incidence of 3% or lesser in either treatment group.

Data of adverse events from Cassier et al. were from Cycle 5-8 in the study, when the patients were combined with doxorubicin.

#### 3.3.1 Hematologic abnormalities

The incidence of neutropenia, leukopenia, and febrile neutropenia were generally higher with docetaxel than with paclitaxel. Compared to docetaxel, Cassier and Swain both reported a higher rate of anemia with paclitaxel (0% vs. 1.3%, Cassier et al.; 0.2% vs. 1.6%, Swain et al.).

#### 3.3.2 Non-hematologic abnormalities

##### 3.3.2.1 Edema

Four of the five trials reported grade 3/4 edema; among these, three trials reported a lower incidence of grade 3/4 edema with paclitaxel than with docetaxel (Jones et al., 0.5% vs. 6.8%; Cassier et al., 0% vs. 2.8%; Watanabe et al., 0% vs. 12.6%). However, Kelly et al. reported a comparable incidence of grade 3/4 edema among two taxanes (0.3% vs. 0%). Overall, the incidence of grade 3/4 edema ranged from 0% to 0.5% wit paclitaxel, and 2.8% to 12.6% with docetaxel, indicating that peripheral edema was one of the more prominent side effects for docetaxel compared to paclitaxel.

##### 3.3.2.2 Peripheral neuropathy

The incidence of peripheral neuropathy was generally low. Cassier et al. reported a higher rate of peripheral neuropathy with paclitaxel than with docetaxel (7.8% with paclitaxel vs. 0% with docetaxel). Kelly et al. reported a slightly higher rate of peripheral neuropathy with docetaxel (4.8%) than with paclitaxel (2.4%).

Kelly and Watanabe both reported higher incidences of motor neuropathy with docetaxel than with paclitaxel (2.3% vs. 5.0%, 0.4% vs. 1.3%, respectively). Jones et al. reported a higher incidence of sensory neuropathy with docetaxel (7.2% with docetaxel vs. 4.1% with paclitaxel), while Watanabe reported a higher incidence rate of sensory with paclitaxel (5.7% with paclitaxel vs. 3.8% with docetaxel).

Overall, the incidence of neuropathy was around 5% in most trials, and there was no consistent trend in favor of a given taxane in terms of occurrence.

##### 3.3.2.3 Skin disorders and mucositis

Jones et al. and Kelly et al. showed low incidences of skin disorders with both paclitaxel and docetaxel (< 5%). The rate of mucositis was low and comparable between the two taxanes, except in Jones trial showing a higher incidence in the docetaxel group (10.8%).

##### 3.3.2.4 Biochemical abnormalities regarding liver function

Watanabe et al. demonstrated a low rate of liver enzyme elevation in both groups (paclitaxel, 3.1%; docetaxel, 0.4%).

##### 3.3.2.5 Gastrointestinal disorders

The docetaxel group had a slightly higher rate of gastrointestinal disorders than the paclitaxel group. Jones et al. reported a rate of 5.4% for both nausea and diarrhea in the docetaxel group versus 2.7% and 0.5% in the paclitaxel group, respectively. Swain et al. reported a rate of 6.8% for diarrhea in the docetaxel group versus 2.2% in the paclitaxel group. All trials reported low incidence rates of grade 3/4 vomiting and constipation for both taxane groups (less than 5% in all groups)

##### 3.3.2.6 Pneumonitis

Drug induced pneumonitis, usually in the form of interstitial pneumonitis, has been associated with taxene use in multiple cancer types (24–26). The incidence of ILD related to docetaxel was estimated around 4.6% (26). In metastatic breast cancer studies, in the CALGB9840 trial which investigated weekly versus tri-weekly paclitaxel, the exact prevalence of pneumonitis was not reported, however grade 3/4 dyspnea was reported to be around 5-8% (27). In this study, 2 patients were reported to die of pneumonia (27). In the five phase III trials discussed in our study, only 1 study (22) reported 1 case of fatal pneumonitis on treatment (TAC regimen, docetaxel, doxorubicin and cyclophosphamide). In general, taxene associated pneumonitis has a relatively rare incidence in the breast cancer trials being reviewed, but is still potentially fatal and should be cautioned in patients receiving taxene treatment.

## 4 Discussion

Taxanes have been shown to significantly improve survival in patients with metastatic and adjuvant breast cancer. However, side effects caused by taxanes may lead to treatment discontinuation and impact treatment outcomes (28). Optimal management of AEs is important to optimize compliance and make sure treatment is completed as planned. In this sense, a thorough understanding of taxanes and their side effects is essential for better management for breast cancer patients. In this review, we carefully compared paclitaxel with docetaxel from reported clinical trials.

Docetaxel seemed associated with slightly higher incidences of grade 3/4 AEs, including hematologic disorders, edema, skin toxicities, and mucositis; while peripheral neuropathy was slightly higher for paclitaxel in most studies. The incidence of other AEs, such as myalgia, was comparable between docetaxel and paclitaxel.

Hematologic AEs are often dose-limiting and may be associated with neutropenic complications such as febrile neutropenia. Jones et al. noted a surprisingly high incidence rate of neutropenia (93.3%) in the docetaxel group. Such finding may be attributed to the relatively high dose of docetaxel and the patient characteristics. In this trial, docetaxel was given at a dose of 100 mg/m^2^ Q3W. Previous studies reported that neutropenia was dose-dependent, infusion-dependent, and was influenced by previous chemotherapy regimen used (4, 29–31). Prior myelotoxic therapy extent might determine the severity of neutropenia. In cancer, neutrophils are also often associated with myeloid-derived suppressor cells (MDSCs), which contribute to an immunosuppressive microenvironment (32). The findings that, in solid tumors and hematologic malignancies, including patients with breast cancer, grade ≥ 3 neutropenia or leukopenia during chemotherapy is consistently associated with improved overall survival, may also reflect the role of neutrophils in promoting cancer progression, although the effects of increased cytotoxic drug exposure should also be taken into consideration (33, 34). The greater incidence of grade ≥ 3 neutropenia with docetaxel compared with paclitaxel may explain the greater survival benefit with docetaxel compared to paclitaxel when both taxanes are given in similar conditions of administration (5). This is still speculation, and whether the onset or length of neutropenia can be prognostic still remains to be validated in further studies (35). The use of prophylactic G-CSF has been shown to drastically reduce the risk of neutropenic complications and shortens the hospitalization stay days in patients with chemotherapy-related myelosuppression (36–38) without impairing overall survival (39).

In this review, we found that the incidence rate of peripheral neuropathy, regardless of its origin (sensory or motor), was less than 10%. Jones et al. prespecified a taxanes dose reduction by 25% in patients experiencing grade 2 or greater neurotoxicity. The average number of treatment cycles for developing grade 2 or greater toxicity was around 4 cycles for both taxanes (371 mg/m^2^ for docetaxel prescribed at 100 mg/m^2^ per cycle and 715 mg/m^2^ for paclitaxel at 175 mg/m^2^). Peripheral neuropathy caused by paclitaxel and docetaxel has been reported to be dose- and time-dependent (4, 36, 40). It has been suggested that Cremophor EL, the solvent of paclitaxel, might contribute to ganglionopathy, axonopathy, and demyelination (4). Several studies have investigated possible drugs to alleviate the neurotoxicity, including tricyclic antidepressants and gabapentin, but all failed to show significant benefits (41–43). Pabst et al. indicated that peripheral neuropathy might impact the economic status of the patients. Other factors related to taxane-induced peripheral neuropathy (TIPN) included diabetes mellitus, overweight, alcohol use (15).

Less than 1% of the paclitaxel group developed grade 3/4 edema, compared to around 10% for the docetaxel group. In the past two decades, several premedication strategies aiming to reduce the rate of edema were developed. Previous studies show that the rate of fluid retention was 64.1% and serious fluid retention was 6.5%, both under the label-recommended dexamethasone 8 mg BID for three days (36). Behar et al. and Semb et al. reported that protein retention by capillary endothelium and drug-induced capillary loss could be a potential explanation (44, 45). Clinically, the median cumulative dose of docetaxel for developing fluid retention, regardless of severity, has been reported as high as 819 mg/m^2^, although 301 mg/m^2^ and 247 mg/m^2^ were also reported (45, 46). It has been suggested as a possible concern that standard 3-day dexamethasone premedication might lead to poor adherence and a higher incidence of infection caused by higher dose of dexamethasone use, which led to several new dexamethasone premedication strategies proposed. Chouhan et al. reported that the use of single intravenous 20-mg dexamethasone might be effective in reducing the rate of all-grade edema to 12.2% (47). Yoo et al. noted that a higher dose of dexamethasone did not significantly increase the infection rate (46). Diuretics may also be useful for the treatment of the fluid retention caused by taxanes (48).

Hypersensitivity reaction (HSR) is another commonly encountered adverse effect for taxane administration. Several studies have speculated that solvents used in taxanes might lead to these undesired side effects (4, 19, 49), and that solvents for paclitaxel can lead to neurotoxicity (4). Markman et al. reported a rate of 9% in hypersensitivity for the paclitaxel group while the manufacturing label described the incidence rate of 15.2% overall and 2.2% in serious hypersensitivity (50). Bookman et al. reported that a short course of glucocorticoids might reduce the rate of all-grade hypersensitivity to 4.6% (51). SC5b-9, a target protein for complement activation involving HSR, was reported to be slightly higher in the paclitaxel group than in the docetaxel group *in vitro* (19). SC5b-9 decreased significantly if pre-treated with dexamethasone and cimetidine (49). This is consistent with the commonly used premedication including both anti-histamine and glucocorticoid for paclitaxel to prevent hypersensitivity. It must be noted, however, preclinical studies have suggested that cimetidine may enhance delayed hypersensitivity in an animal model of burn injury (52). The clinical implications remain to be clarified whether cimetidine will adversely induce hypersensitivity in taxene treatment, and whether this is a class effect that involves other antihistamines or only to this drug alone.

In current clinical practice, paclitaxel is frequently administered weekly (QW), while docetaxel is usually given every three weeks (Q3W), although a 2-weekly administration of docetaxel has been shown to be as effective as a 3-weekly schedule with less hematological toxicity (53) and is recommended by SIOG guidelines for patients who are not fit enough to receive the standard schedule (54). Moreover, in the era of COVID-19, the Q3W dosing schedule offers a more patient-friendly regimen, which minimizes hospital visits and thus decreases the risk of infection. A study conducted by Early Breast Cancer Trialists’ Collaborative Group (EBCTCG) demonstrated the importance of adherence to chemotherapeutic agents especially for women aged less than 50 years old (55). This study highlights the advantages of a longer interval for treatment as well. In this scenario, docetaxel given in Q3W would be a more appealing treatment option, which may potentially improve the treatment compliance and adherence in the COVID-19 era.

There are three major limitations in our study. First of all, our review was unable to extract the safety information of single taxane treatment because the combinational therapy, which has been frequently shown to be superior to the monotherapy in the treatment of mBC (56, 57), was commonly used in most trials. Furthermore, few trials directly compared paclitaxel to docetaxel head to head. For example, PERUSE trial reported that paclitaxel and nab-paclitaxel could be substituted for docetaxel in the pertuzumab-trastuzumab-taxane regimen for HER2 mBC patients. However, we failed to retrieve specific grade 3/4 AEs event rate of either nab-paclitaxel, docetaxel, or nab-paclitaxel from the article. Secondly, patient compliance and patient-reported outcomes (PRO) were not discussed in our analysis; however, these aspects reflect the tolerability of taxane and are highly associated with drug’s safety. The recent discovery of the difference between patient-reported and physician-reported outcomes highlights the importance of PRO, which is considered to be valuable and clinically informative for treatment (58). Our review could not assess the patient’s quality of life (QoL), which is a more holistic surrogate for patient’s general well-being and tolerability. Also, detailed and quantitative QoL measurements were not commonly reported in earlier trials. This illuminated the further need for exploring the PROs between the taxanes.

In summary, taxanes are well-tolerated and active chemotherapy regimens for treatment in breast cancer, and after nearly two decades of widespread use, a considerable amount of experience has been accumulated. Our review highlights the common features for taxane-related side effects and provides further insights into how to better prevent and manage AEs. In our study, we reviewed grade 3/4 AEs associated with taxane-containing regimens. Our research concluded that most AEs are manageable. Grade 3/4 neutropenia is more frequently reported with docetaxel, but this side effect is commonly associated with a greater anti-tumor activity and can be easily prevented by prophylactic G-CSF use. We also highlight the importance of dexamethasone premedication strategies which are important to prevent the risk of fluid retention. One specific AE that addresses concern is peripheral neuropathy because it cumulative and can sometimes be irreversible. Further research for preventing neurotoxicity and investigating its optimal management are urgently needed.

## Data availability statement

The raw data supporting the conclusions of this article will be made available by the authors, without undue reservation.

## Author contributions

J-IL and T-CC developed the concept. J-IL searched and reviewed the literatures, captured and analyzed the data, and wrote the first draft of the manuscript. T-CC and C-YL provided critical revision of the manuscript for important intellectual content. T-CC, C-YL, and J-IL jointly developed the structure and arguments of the paper. J-IL and T-CC made critical revisions to the paper. All authors contributed to the article and approved the submitted version.

## Funding

This systemic review was supported by Taiwan Clinical Oncology Research Foundation and the Melissa Lee Cancer Foundation (MLCF-V111_A11102).

## Acknowledgments

The authors acknowledge Lulu Hou and Alice Tsai from Sanofi Taiwan Co., Ltd provided writing and editorial assistance in the preparation of this manuscript; and Ellie Huang from Formosa Biomedical Technology Corp. CRO Division in the statistical analysis and editorial support, under the direction of the authors. The authors were responsible for all content and editorial decisions, and received no honoraria related to the development of this publication.

## Conflict of interest

The authors declare that the research was conducted in the absence of any commercial or financial relationships that could be construed as a potential conflict of interest.

## Publisher’s note

All claims expressed in this article are solely those of the authors and do not necessarily represent those of their affiliated organizations, or those of the publisher, the editors and the reviewers. Any product that may be evaluated in this article, or claim that may be made by its manufacturer, is not guaranteed or endorsed by the publisher.

## References

[1] De Laurentiis M Cancello G D'Agostino D Giuliano M Giordano A Montagna E . Taxane-based combinations as adjuvant chemotherapy of early breast cancer: A meta-analysis of randomized trials. J Clin Oncol (2008) 26(1):44–53. doi: 10.1200/jco.2007.11.3787 18165639

[2] Ferguson T Wilcken N Vagg R Ghersi D Nowak AK . Taxanes for adjuvant treatment of early breast cancer. Cochrane Database Syst Rev (2007) 4):Cd004421. doi: 10.1002/14651858.CD004421.pub2 17943815

[3] Ghoncheh M Pournamdar Z Salehiniya H . Incidence and mortality and epidemiology of breast cancer in the world. Asian Pac J Cancer Prev (2016) 17(S3):43–6. doi: 10.7314/apjcp.2016.17.s3.43 27165206

[4] Marupudi NI Han JE Li KW Renard VM Tyler BM Brem H . Paclitaxel: A review of adverse toxicities and novel delivery strategies. Expert Opin Drug Saf (2007) 6(5):609–21. doi: 10.1517/14740338.6.5.609 17877447

[5] Jones SE Erban J Overmoyer B Budd GT Hutchins L Lower E . Randomized phase iii study of docetaxel compared with paclitaxel in metastatic breast cancer. J Clin Oncol (2005) 23(24):5542–51. doi: 10.1200/jco.2005.02.027 16110015

[6] Company B-MS . Taxol® (Paclitaxel) injection (2011). Available at: https://www.accessdata.fda.gov/drugsatfda_docs/label/2011/020262s049lbl.pdf.

[7] Henderson IC Berry DA Demetri GD Cirrincione CT Goldstein LJ Martino S . Improved outcomes from adding sequential paclitaxel but not from escalating doxorubicin dose in an adjuvant chemotherapy regimen for patients with node-positive primary breast cancer. J Clin Oncol (2003) 21(6):976–83. doi: 10.1200/jco.2003.02.063 12637460

[8] Swain SM Jeong JH Geyer CE Jr. Costantino JP Pajon ER Fehrenbacher L . Longer therapy, iatrogenic amenorrhea, and survival in early breast cancer. N Engl J Med (2010) 362(22):2053–65. doi: 10.1056/NEJMoa0909638 PMC2935316 20519679

[9] Seidman AD Tiersten A Hudis C Gollub M Barrett S Yao TJ . Phase ii trial of paclitaxel by 3-hour infusion as initial and salvage chemotherapy for metastatic breast cancer. J Clin Oncol (1995) 13(10):2575–81. doi: 10.1200/jco.1995.13.10.2575 7595709

[10] Perez EA Vogel CL Irwin DH Kirshner JJ Patel R . Multicenter phase ii trial of weekly paclitaxel in women with metastatic breast cancer. J Clin Oncol (2001) 19(22):4216–23. doi: 10.1200/jco.2001.19.22.4216 11709565

[11] Swain SM Baselga J Kim SB Ro J Semiglazov V Campone M . Pertuzumab, trastuzumab, and docetaxel in Her2-positive metastatic breast cancer. N Engl J Med (2015) 372(8):724–34. doi: 10.1056/NEJMoa1413513 PMC5584549 25693012

[12] Bachelot T Ciruelos E Schneeweiss A Puglisi F Peretz-Yablonski T Bondarenko I . Preliminary safety and efficacy of first-line pertuzumab combined with trastuzumab and taxane therapy for Her2-positive locally recurrent or metastatic breast cancer (Peruse). Ann Oncol (2019) 30(5):766–73. doi: 10.1093/annonc/mdz061 30796821

[13] Pegram MD Konecny GE O'Callaghan C Beryt M Pietras R Slamon DJ . Rational combinations of trastuzumab with chemotherapeutic drugs used in the treatment of breast cancer. J Natl Cancer Inst (2004) 96(10):739–49. doi: 10.1093/jnci/djh131 15150302

[14] Di Modica M Sfondrini L Regondi V Varchetta S Oliviero B Mariani G . Taxanes enhance trastuzumab-mediated adcc on tumor cells through Nkg2d-mediated nk cell recognition. Oncotarget (2016) 7(1):255–65. doi: 10.18632/oncotarget.6353 PMC4807996 26595802

[15] Pabst L Velten M Fischbach C Kalish M Pflumio C Pivot X . Persistent taxane-induced neuropathy in elderly patients treated for localized breast cancer. Breast J (2020) 26(12):2376–82. doi: 10.1111/tbj.14123 33307596

[16] Kramer JA Curran D Piccart M de Haes JC Bruning PF Klijn JG . Randomised trial of paclitaxel versus doxorubicin as first-line chemotherapy for advanced breast cancer: Quality of life evaluation using the eortc qlq-C30 and the Rotterdam symptom checklist. Eur J Cancer (2000) 36(12):1488–97. doi: 10.1016/s0959-8049(00)00134-9 10930796

[17] Cassier PA Chabaud S Trillet-Lenoir V Peaud PY Tigaud JD Cure H . A phase-iii trial of doxorubicin and docetaxel versus doxorubicin and paclitaxel in metastatic breast cancer: Results of the erasme 3 study. Breast Cancer Res Treat (2008) 109(2):343–50. doi: 10.1007/s10549-007-9651-3 17611792

[18] Piccart MJ Klijn J Paridaens R Nooij M Mauriac L Coleman R . Corticosteroids significantly delay the onset of docetaxel-induced fluid retention: Final results of a randomized study of the European organization for research and treatment of cancer investigational drug branch for breast cancer. J Clin Oncol (1997) 15(9):3149–55. doi: 10.1200/jco.1997.15.9.3149 9294478

[19] Weiszhár Z Czúcz J Révész C Rosivall L Szebeni J Rozsnyay Z . Complement activation by polyethoxylated pharmaceutical surfactants: Cremophor-El, tween-80 and tween-20. Eur J Pharm Sci (2012) 45(4):492–8. doi: 10.1016/j.ejps.2011.09.016 21963457

[20] Schwartz JR . Dexamethasone premedication for prophylaxis of taxane toxicities: Can the doses be reduced when paclitaxel or docetaxel are given weekly? J Oncol Pharm Pract (2012) 18(2):250–6. doi: 10.1177/1078155211409473 21807762

[21] Kelly CM Green MC Broglio K Thomas ES Brewster AM Valero V . Phase iii trial evaluating weekly paclitaxel versus docetaxel in combination with capecitabine in operable breast cancer. J Clin Oncol (2012) 30(9):930–5. doi: 10.1200/jco.2011.36.2079 22331946

[22] Swain SM Tang G Geyer CE Jr. Rastogi P Atkins JN Donnellan PP . Definitive results of a phase iii adjuvant trial comparing three chemotherapy regimens in women with operable, node-positive breast cancer: The nsabp b-38 trial. J Clin Oncol (2013) 31(26):3197–204. doi: 10.1200/jco.2012.48.1275 PMC3757290 23940225

[23] Watanabe T Kuranami M Inoue K Masuda N Aogi K Ohno S . Comparison of an Ac-taxane versus Ac-free regimen and paclitaxel versus docetaxel in patients with lymph node-positive breast cancer: Final results of the national surgical adjuvant study of breast cancer 02 trial, a randomized comparative phase 3 study. Cancer (2017) 123(5):759–68. doi: 10.1002/cncr.30421 PMC6668007 28081304

[24] Harries M Moss C Perren T Gore M Hall G Everard M . A phase II feasibility study of carboplatin followed by sequential weekly paclitaxel and gemcitabine as first-line treatment for ovarian cancer. Br J Cancer (2004) 91(4):627. doi: 10.1038/sj.bjc.6602000 15238984 PMC2364776

[25] Graziano SL Herndon JE 2nd Socinski MA Wang X Watson D Vokes E . Phase II trial of weekly dose-dense paclitaxel in extensive-stage small cell lung cancer: Cancer and leukemia group b study 39901. J Thorac Oncol (2008) 3(2):158. doi: 10.1097/JTO.0b013e318161225e 18303437

[26] Tamiya A Naito T Miura S Morii S Tsuya A Nakamura Y . Interstitial lung disease associated with docetaxel in patients with advanced non-small cell lung cancer. Anticancer Res (2012) 32(3):1103–6. 22399640

[27] Seidman AD Berry D Cirrincione C Harris L Muss H Marcom PK . Randomized phase III trial of weekly compared with every-3-weeks paclitaxel for metastatic breast cancer, with trastuzumab for all HER-2 overexpressors and random assignment to trastuzumab or not in HER-2 nonoverexpressors: Final results of cancer and leukemia group b protocol 9840. J Clin Oncol (2008) 26(10):1642. doi: 10.1200/JCO.2007.11.6699 18375893

[28] Schwentner L Van Ewijk R Kühn T Flock F Felberbaum R Blettner M . Exploring patient- and physician-related factors preventing breast cancer patients from guideline-adherent adjuvant chemotherapy-results from the prospective multi-center study Brenda ii. Support Care Cancer (2016) 24(6):2759–66. doi: 10.1007/s00520-016-3088-3 26816089

[29] Sparano JA Wang M Martino S Jones V Perez EA Saphner T . Weekly paclitaxel in the adjuvant treatment of breast cancer. N Engl J Med (2008) 358(16):1663–71. doi: 10.1056/NEJMoa0707056 PMC2743943 18420499

[30] Stemmler HJ Harbeck N Gröll de Rivera I Vehling Kaiser U Rauthe G Abenhardt W . Prospective multicenter randomized phase iii study of weekly versus standard docetaxel (D2) for first-line treatment of metastatic breast cancer. Oncology (2010) 79(3-4):197–203. doi: 10.1159/000320640 21358207

[31] Schröder CP de Munck L Westermann AM Smit WM Creemers GJ de Graaf H . Weekly docetaxel in metastatic breast cancer patients: No superior benefits compared to three-weekly docetaxel. Eur J Cancer (2011) 47(9):1355–62. doi: 10.1016/j.ejca.2010.12.018 21251813

[32] Wu L Saxena S Awaji M Singh RK . Tumor-associated neutrophils in cancer: Going pro. Cancers (Basel) (2019) 11(4):564. doi: 10.3390/cancers11040564 PMC6520693 31010242

[33] Shitara K Matsuo K Oze I Mizota A Kondo C Nomura M . Meta-analysis of neutropenia or leukopenia as a prognostic factor in patients with malignant disease undergoing chemotherapy. Cancer Chemother Pharmacol (2011) 68(2):301–7. doi: 10.1007/s00280-010-1487-6 20960191

[34] Kasi PM Grothey A . Chemotherapy-induced neutropenia as a prognostic and predictive marker of outcomes in solid-tumor patients. Drugs (2018) 78(7):737–45. doi: 10.1007/s40265-018-0909-3 29754293

[35] Chen Y Shi Y Yan H Rong Wang Y Hai Dai G . Timing of chemotherapy-induced neutropenia: The prognostic factor in advanced pancreatic cancer patients treated with gemcitabine / gemcitabine-based chemotherapy. Oncotarget (2017) 8(39):66593–600. doi: 10.18632/oncotarget.16980 PMC5630440 29029540

[36] Ho MY Mackey JR . Presentation and management of docetaxel-related adverse effects in patients with breast cancer. Cancer Manag Res (2014) 6:253–9. doi: 10.2147/cmar.S40601 PMC4041377 24904223

[37] Martín M Lluch A Seguí MA Ruiz A Ramos M Adrover E . Toxicity and health-related quality of life in breast cancer patients receiving adjuvant docetaxel, doxorubicin, cyclophosphamide (Tac) or 5-fluorouracil, doxorubicin and cyclophosphamide (Fac): Impact of adding primary prophylactic granulocyte-colony stimulating factor to the tac regimen. Ann Oncol (2006) 17(8):1205–12. doi: 10.1093/annonc/mdl135 16766587

[38] Smith TJ Khatcheressian J Lyman GH Ozer H Armitage JO Balducci L . 2006 Update of recommendations for the use of white blood cell growth factors: An evidence-based clinical practice guideline. J Clin Oncol (2006) 24(19):3187–205. doi: 10.1200/jco.2006.06.4451 16682719

[39] Meisel A von Felten S Vogt DR Liewen H de Wit R de Bono J . Severe neutropenia during cabazitaxel treatment is associated with survival benefit in men with metastatic castration-resistant prostate cancer (Mcrpc): A *Post-hoc* analysis of the tropic phase iii trial. Eur J Cancer (2016) 56:93–100. doi: 10.1016/j.ejca.2015.12.009 26829012

[40] Akerley W 3rd . Paclitaxel in advanced non-small cell lung cancer : An alternative high-dose weekly schedule. Chest (2000) 117(4 Suppl 1):152s–5s. doi: 10.1378/chest.117.4_suppl_1.152s 10777471

[41] Lee JJ Swain SM . Peripheral neuropathy induced by microtubule-stabilizing agents. J Clin Oncol (2006) 24(10):1633–42. doi: 10.1200/jco.2005.04.0543 16575015

[42] Rivera E Mejia JA Arun BK Adinin RB Walters RS Brewster A . Phase 3 study comparing the use of docetaxel on an every-3-Week versus weekly schedule in the treatment of metastatic breast cancer. Cancer (2008) 112(7):1455–61. doi: 10.1002/cncr.23321 18300256

[43] Rao RD Michalak JC Sloan JA Loprinzi CL Soori GS Nikcevich DA . Efficacy of gabapentin in the management of chemotherapy-induced peripheral neuropathy: A phase 3 randomized, double-blind, placebo-controlled, crossover trial (N00c3). Cancer (2007) 110(9):2110–8. doi: 10.1002/cncr.23008 17853395

[44] Béhar A Pujade-Lauraine E Maurel A Brun MD Chauvin FF Feuilhade de Chauvin F . The pathophysiological mechanism of fluid retention in advanced cancer patients treated with docetaxel, but not receiving corticosteroid comedication. Br J Clin Pharmacol (1997) 43(6):653–8. doi: 10.1046/j.1365-2125.1997.00613.x PMC2042780 9205828

[45] Semb KA Aamdal S Oian P . Capillary protein leak syndrome appears to explain fluid retention in cancer patients who receive docetaxel treatment. J Clin Oncol (1998) 16(10):3426–32. doi: 10.1200/jco.1998.16.10.3426 9779722

[46] Yoo KE Kang RY Lee JY Lee YJ Suh SY Kim KS . Awareness of the adverse effects associated with prophylactic corticosteroid use during docetaxel therapy. Support Care Cancer (2015) 23(7):1969–77. doi: 10.1007/s00520-014-2547-y 25500718

[47] Chouhan JD Herrington JD . Single premedication dose of dexamethasone 20 mg iv before docetaxel administration. J Oncol Pharm Pract (2011) 17(3):155–9. doi: 10.1177/1078155210367950 20447949

[48] Singer EA Srinivasan R . Intravenous therapies for castration-resistant prostate cancer: Toxicities and adverse events. Urol Oncol (2012) 30(4 Suppl):S15–9. doi: 10.1016/j.urolonc.2011.09.003 PMC3274605 22014836

[49] Wang H Cheng G Du Y Ye L Chen W Zhang L . Hypersensitivity reaction studies of a polyethoxylated castor oil-free, liposome-based alternative paclitaxel formulation. Mol Med Rep (2013) 7(3):947–52. doi: 10.3892/mmr.2013.1264 PMC3597461 23291923

[50] Markman M Kennedy A Webster K Kulp B Peterson G Belinson J . Paclitaxel-associated hypersensitivity reactions: Experience of the gynecologic oncology program of the Cleveland clinic cancer center. J Clin Oncol (2000) 18(1):102–5. doi: 10.1200/jco.2000.18.1.102 10623699

[51] Bookman MA Kloth DD Kover PE Smolinski S Ozols RF . Short-course intravenous prophylaxis for paclitaxel-related hypersensitivity reactions. Ann Oncol (1997) 8(6):611–4. doi: 10.1023/a:1008207025430 9261533

[52] Jafarzadeh 1 A Nemati M Rezayati MT Ebrahimi M Hassan ZM . Cimetidine enhances delayed-type hypersensitivity responses and serum interleukin (IL)-2, -10, -12, and IL-17 levels after burn injury in an animal model. J Immunotoxicol (2013) 10(2):201–9. doi: 10.3109/1547691X.2012.708365 22946962

[53] Kellokumpu-Lehtinen PL Harmenberg U Joensuu T McDermott R Hervonen P Ginman C . 2-weekly versus 3-weekly docetaxel to treat castration-resistant advanced prostate cancer: A randomised, phase 3 trial. Lancet Oncol (2013) 14(2):117–24. doi: 10.1016/s1470-2045(12)70537-5 23294853

[54] Droz JP Aapro M Balducci L Boyle H Van den Broeck T Cathcart P . Management of prostate cancer in older patients: Updated recommendations of a working group of the international society of geriatric oncology. Lancet Oncol (2014) 15(9):e404–14. doi: 10.1016/s1470-2045(14)70018-x 25079103

[55] Early Breast Cancer Trialists’ Collaborative Group . Polychemotherapy for early breast cancer: An overview of the randomised trials. early breast cancer trialists' collaborative group. Lancet (1998) 352(9132):930–42. 9752815

[56] O'Shaughnessy J Miles D Vukelja S Moiseyenko V Ayoub JP Cervantes G . Superior survival with capecitabine plus docetaxel combination therapy in anthracycline-pretreated patients with advanced breast cancer: Phase iii trial results. J Clin Oncol (2002) 20(12):2812–23. doi: 10.1200/jco.2002.09.002 12065558

[57] Albain KS Nag SM Calderillo-Ruiz G Jordaan JP Llombart AC Pluzanska A . Gemcitabine plus paclitaxel versus paclitaxel monotherapy in patients with metastatic breast cancer and prior anthracycline treatment. J Clin Oncol (2008) 26(24):3950–7. doi: 10.1200/jco.2007.11.9362 18711184

[58] Nyrop KA Deal AM Reeder-Hayes KE Shachar SS Reeve BB Basch E . Patient-reported and clinician-reported chemotherapy-induced peripheral neuropathy in patients with early breast cancer: Current clinical practice. Cancer (2019) 125(17):2945–54. doi: 10.1002/cncr.32175 31090930

